# Small extracellular vesicles promote cell survival and neuritogenesis in vitro in a manner dependent on dosage and cell of origin

**DOI:** 10.1038/s41598-026-53763-2

**Published:** 2026-06-03

**Authors:** Matyas Kutnyanszky, Phil Stephens, Ben Mead

**Affiliations:** 1 School of Optometry and Vision Sciences, Cardiff University, Cardiff, CF24 4HQ UK; 2 School of Dentistry, Cardiff University, Cardiff, CF14 4XY UK

**Keywords:** Cell biology, Neuroscience, Stem cells

## Abstract

Mesenchymal Stem Cells (MSC) possess a diverse secretome with well-established neuroprotective effects. Form among the materiel released by these cells, extracellular vesicles (EVs) have gained particular interest lately, owing to their good safety profile, stability, and relative ease of use as a cell-free therapy. These lipid-enclosed nano-carriers can significantly alter the survival of recipient cells through the delivery of a wide variety of signalling molecules, the exact composition of which is highly dependent on the type, age, and environment of the donor cells. Glaucoma is a chronic progressive optic neuropathy characterised by the loss of Retinal Ganglion Cells whose axons make up the optic nerve. Preservation of these neurons via the administration of the right EVs represents a promising approach for slowing disease progression, thereby preventing vision loss. Here, we evaluate and compare the protective and neuritogenic potential of small extracellular vesicles (sEVs), a subset of EVs with a diameter smaller than 220 nm, from six different cell types using a rodent *in vitro* model of RGC degeneration. Our findings showed that Adipose Mesenchymal Stem Cells release the most potent sEVs, with Bone Marrow being a close second. EVs released by cells of the Umbilical Cord, Dental Pulp, Dermal Fibroblasts, and an Oral Mucosal Lamina Propria-Progenitor cells did not have an observable benefit. Thus, our study provides greater insight into how the efficacy of different EVs compares to each other.

## Introduction

Glaucoma refers to a set of ocular diseases characterised by the gradual loss of Retinal Ganglion Cells (RGCs), the neurons responsible for relaying visual stimuli to the brain through their axons, which make up the optic nerve^1,2^. The retina, on account of being an outgrowth of the central nervous system, possesses minimal regenerative capabilities^3^. Thus, the loss of these neurons and the subsequent ablation in eye-to-brain communication lead to significant and permanent visual impairment. Indeed, Glaucoma is currently considered the leading cause of irreversible blindness worldwide^4,5^.

Mesenchymal Stem Cells (MSCs) are stromal cells capable of self-renewal and multilineage differentiation situated in mesenchymal tissues^6,7^. As interest in the field of stem cell-based therapies is rising, more and more attempts are being made to harness the power of MSC for the treatment of various diseases^8–10^. One way MSCs operate is by releasing various neurotrophic and neuroprotective factors in a paracrine fashion, thereby encouraging the survival and regeneration of neighbouring cells^11,12^. Extracellular Vesicles (EVs) are part of this protective secretome, lipid-bound nano-sized particles our body utilises for cell-to-cell communication by transporting proteins, nucleic acids, and other biomolecules^13–16^. Following their formation and loading, these vesicles are released into the intracellular space from where they can be taken up by other cells, altering their gene expression profiles and internal biochemistry^17,18^. EVs are released from almost every cell in our body and can be easily isolated from biological fluids, or conditioned media in the case of *in vitro* cell cultures. Exosomes, which appear to be mostly responsible for the beneficial paracrine effects of MSCs, are a subcategory of EVs that occupy the 30–150 nm size range. Although the lack of specific markers makes the isolation of these smaller vesicles quite difficult, a simple and commonly used approach is filtration with a 220 nm cutoff point, which creates a nanoparticle population referred to as small Extracellular Vesicles (sEVs)^19–21^.

To date, several studies have been published utilising EVs sourced from different MSCs on various *in vivo* and *in vitro* models of ocular diseases^22^. The most well-studied are vesicles isolated from Bone Marrow stem cells, which have been shown to protect trabecular meshwork cells from oxidative stress^23^ and encourage the growth of cortical neuron axons^24^ *in vitro*. When injected intravitreally, these vesicles were able to reduce inflammation and oxidative stress in diabetic retinal injury models^25^ and reduce RGC loss in various animal glaucoma models^13,14^. Similarly, those originating from Adipose or Umbilical Cord stem cells were both successfully used to attenuate RGC death and retinal degeneration in animal models of glaucoma^26–28^ or retinal neuropathy^29–34^, with the latter also achieving similar success *in vitro* on human retinal vascular endothelial cells (HRECs) and rat retinal neurons cultured in hyperglycemic conditions^35,36^.

For the other cell types used here, although the use of EVs from Dental Pulp stem cells in ocular conditions remains underexplored, some studies have already shown intravitreal injection of the stem cells themselves to be neuroprotective in rodent glaucoma models^37,38^, and their EVs to improve gait in a rat Parkinson’s disease model^39^. Unlike MSC-EVs, Fibroblast-derived vesicles have been shown not to be neuroprotective in a wide variety of retinal injury models, and were included to serve as a negative control^13,14,27^. Finally, EVs from an immortalised Oral Mucosal Lamina Propria-Progenitor cells (OMLP-PCs; referred to as OMPCs from now on) were also included for their ability to encourage cell proliferation and wound repopulation in vitro^40^.

Although all of these cell types have demonstrated some degree of success, not all vesicles are created equal. Cargo composition is influenced by several factors, including the type, age, and differentiation stage of the progenitor cells, as well as other internal and external stimuli^19,41,42^. As a result, sEVs of different origins may elicit a varying degree of neuroprotective responses in recipient cells. Addressing this, the present study aimed to identify which of the most commonly used sEVs is best at promoting cell survival and neurite growth. To this end, we isolated vesicles from human Adipose (hADSC), Bone Marrow (hBMSC), Umbilical Cord (hUCSC), and Dental Pulp (hDPSC) stem cells, alongside human Dermal Fibroblasts (hDF) and Oral Progenitors (hOMPC), and compared their efficiency in an *in vitro* model of traumatic nerve damage.

## Results

### Mesenchymal stem cells secrete small vesicles with exosome surface markers

**Fig. 1:**
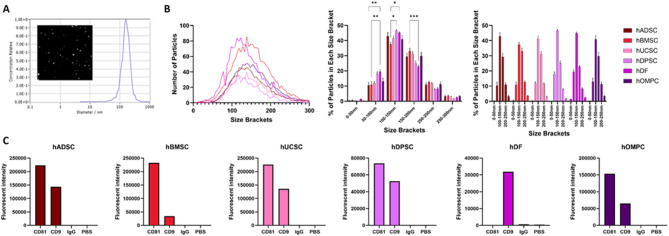
Small Extracellular Vesicle (sEV) secretion by five different Mesenchymal Stem Cells. **(A)** Representative images of ZetaView results showing the size distribution of sEVs. **(B)** Size distribution of sEVs isolated from five different MSCs. Data was compiled from multiple isolations (~8). **(C)** Time-resolved Fluorescence (TRF) of CD81, CD9, and IgG of sEVs samples. hADSC: human Adipose Stem Cell, hBMSC: Human Bone Marrow Stem Cell, hDPSC: human Dental Pulp Stem Cells, hUCSC: human Umbilical Cord Stem Cell, hDF: human Dermal Fibroblast, hOMPC: human Oral Mucosal Progenitor Cell.

All 6 cell types release small Extracellular Vesicles, visualised and measured using a ZetaView Nanoparticle Tracking Analyser and Time-resolved Fluorescence (TRF). Although concentrations varied, the size distribution was similar among vesicles originating from different cell types. Particle sizes ranged between 50 and 300 nm with modes of 130–150 nm for each sample (Fig. 1B). There were a number of observable differences, mostly arising from hDF-sEVs preferentially clustering in the lower size brackets, however, this did not affect which brackets contained the highest and lowest number of particles. Either CD81 or CD9 were detectable in each population, although in different concentrations (Fig. 1C). Notably, hDF-sEVs did not appear to possess CD81 markers. The average number of sEVs isolated from these cell cultures was calculated to be around 3.39 × 10^9^ +/- 5.80 × 10^8^ for hADSC; 3.51 × 10^9^ +/- 3.62 × 10^8^ for hBMSC; 2.74 × 10^10^.

+/- 4.38 × 10^9^ for hUCSC; 1.33 × 10^10^ +/- 7.01 × 10^9^ for hDPSC; 3.30 × 10^10^ +/- 3.79 × 10^9^ for hDF; and 1.05 × 10^10^ +/- 2.72 × 10^9^ for OMPC, expressed as particles per culture flask per media exchange (+/- SEM).

### sEVs released by hADSC and hBMSC improved overall retinal cell survival

**Fig. 2:**
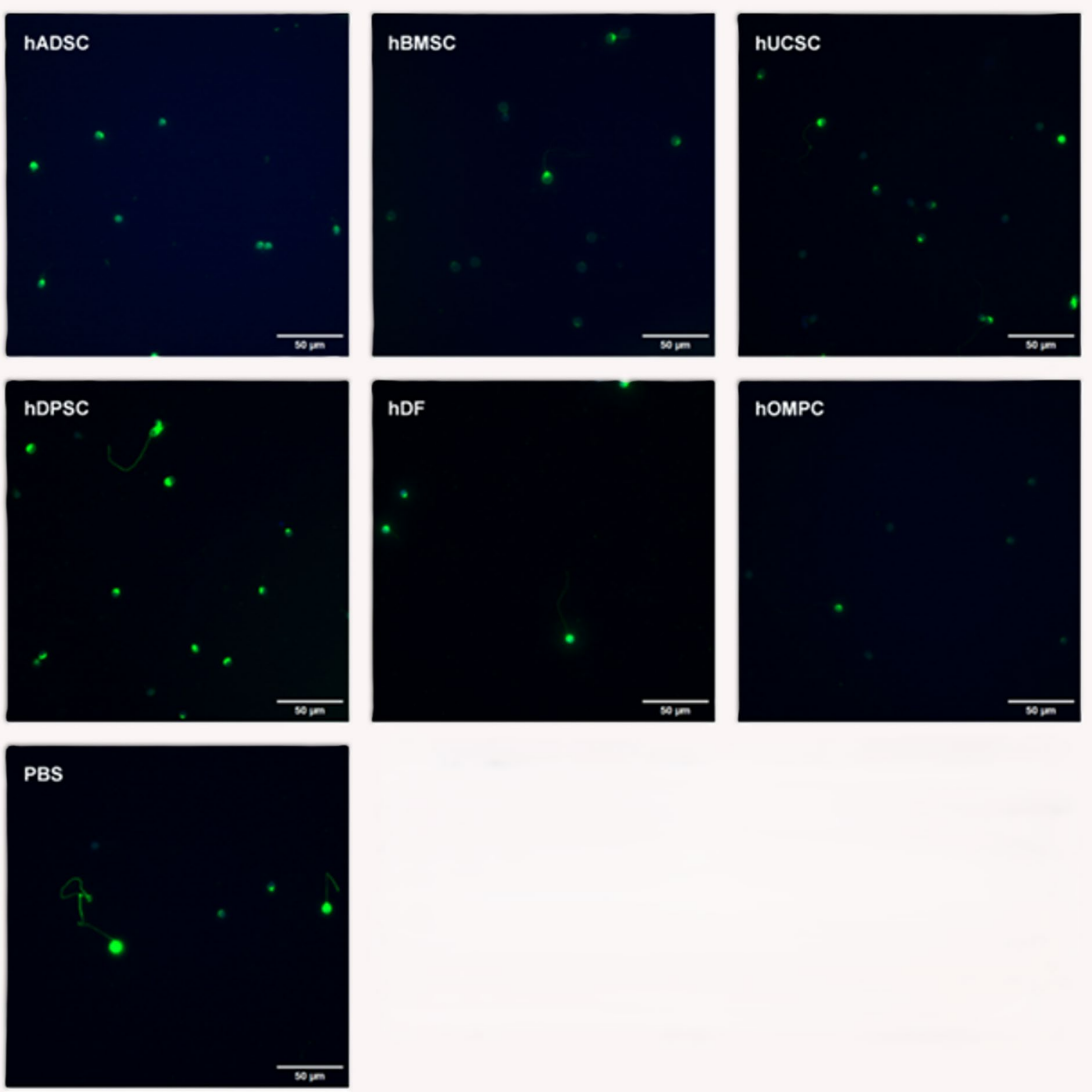
Heterogeneous rat retinal cell cultures treated with 3 × 10^9^ small extracellular vesicles (sEVs). Images are representative of the entire culture. Cells were stained against ßlll-tubulin (green; retinal ganglion cells) and with DAPI (blue). *n*=3 with two technical repeats. Scale bars: 50 µm. hADSC: human Adipose Stem Cell, hBMSC: Human Bone Marrow Stem Cell, hDPSC: human Dental Pulp Stem Cells. hUCSC: human Umbilical Cord Stem Cell, hDF: human Dental Fibroblast hOMPC: human Oral Mucosal progenitor Cell.

**Fig. 3:**
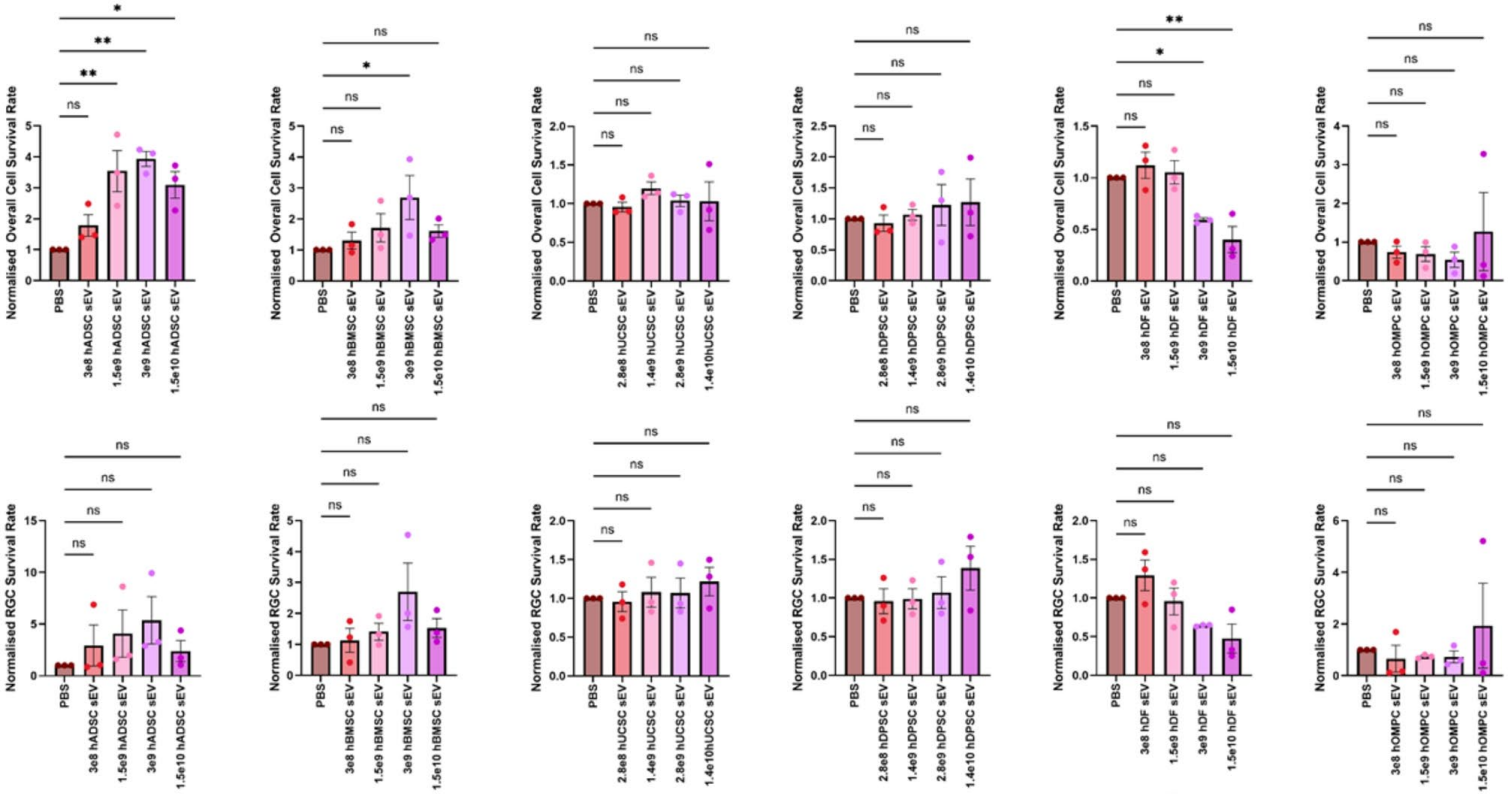
Cell survival in heterogeneous retinal cell cultures treated with small extracellular vesicles (sEVs) from various cell types. Overall cell survival rates (top) and RGC survival rates (bottom) adjusted to PBS controls are shown. Treatment groups are labelled as the number of sEVs per 350 µ1 culture medium with error bars showing the standard error of the mean (*n* = 3). Comparisons were carried out using ANOVA. hADSC: human Adipose Stem Cell, hBMSC: Human Bone Marrow Stem Cell, hDPSC: human Dental Pulp Stem Cells, hUCSC: human Umbilical Cord Stem Cell, hDF: human Dermal Fibroblasts, hOMPC: human Oral Mucosal Progenitor Cell, PBS: Phosphate Buffered Saline ** *p* < 0.01; * *p* < 0.05, ns non-significant.

RGC cultures, isolated rat retinal cells were treated with sEVs, incubated for three days, and stained against β-III-tubulin to survival and neurite growth (Fig. 2). Treatment with either hADSC or hBMSC sEVs successfully increased the number of DAPI-stained cells observed in heterogeneous retinal cell cultures. The best results were achieved with 3 × 10^9^ sEVs, in both cases elevating the number of surviving cells 3.93 +/- 0.24-fold (p: 0.0015) and 2.69 +/- 0.71-fold (p: 0.0466), respectively (Fig. 3). At the higher dose of 1.5 × 10^10^ particles per well, neuroprotection was still observable, albeit to a lesser extent. Vesicles sourced from hUCSC and hDPSC had no observable influence on retinal cell survival, while those originating from hOMPCs appeared to slightly decrease cell survival rates at all concentrations used. Notably, delivering hDF-sEVs at higher doses of 3 × 10^9^ and 1.5 × 10^10^ particles significantly decreased the number of surviving cells, by 0.59 +/- 0.02 fold (p:0.0402) and 0.4 +/- 0.13 fold (p: 0.0041) respectively.

### sEVs released by hADSC and hBMSC improved RGC survival

The number of RGCs (identified by their morphology and βIII-tubulin positive staining) was noticeably higher in wells treated with hADSC or hBMSC sEVs. The best results were again achieved using 3 × 10^9^ vesicles per well, although the differences failed to reach statistical significance. Here, the number of RGC was 5.36 +/- 2.29 (p: 0.2928) times higher for hADSC-sEV treatment and 2.70 +/- 0.93 (p: 0.0965) times higher for hBMSC-sEV treatment compared to PBS-treated controls. hUCSC sEVs did not seem to elicit a significant change in cell numbers, while hDPSC sEVs trended towards an increased survival rate (1.39 +/- 0.28; p: 0.4108) when 1.4 × 10^10^ vesicles were applied. Similarly to overall cell survival rates, hOMPC and hDF sEVs appeared to have a slightly and moderately detrimental effect, respectively, with the most noticeable being 1.5 × 10^10^ hDF sEVs leading to a 0.48 +/- 0.19 fold change (p:0.0856).

### sEVs released by hADSC and hBMSC improved RGC neurite length and number

**Fig. 4:**
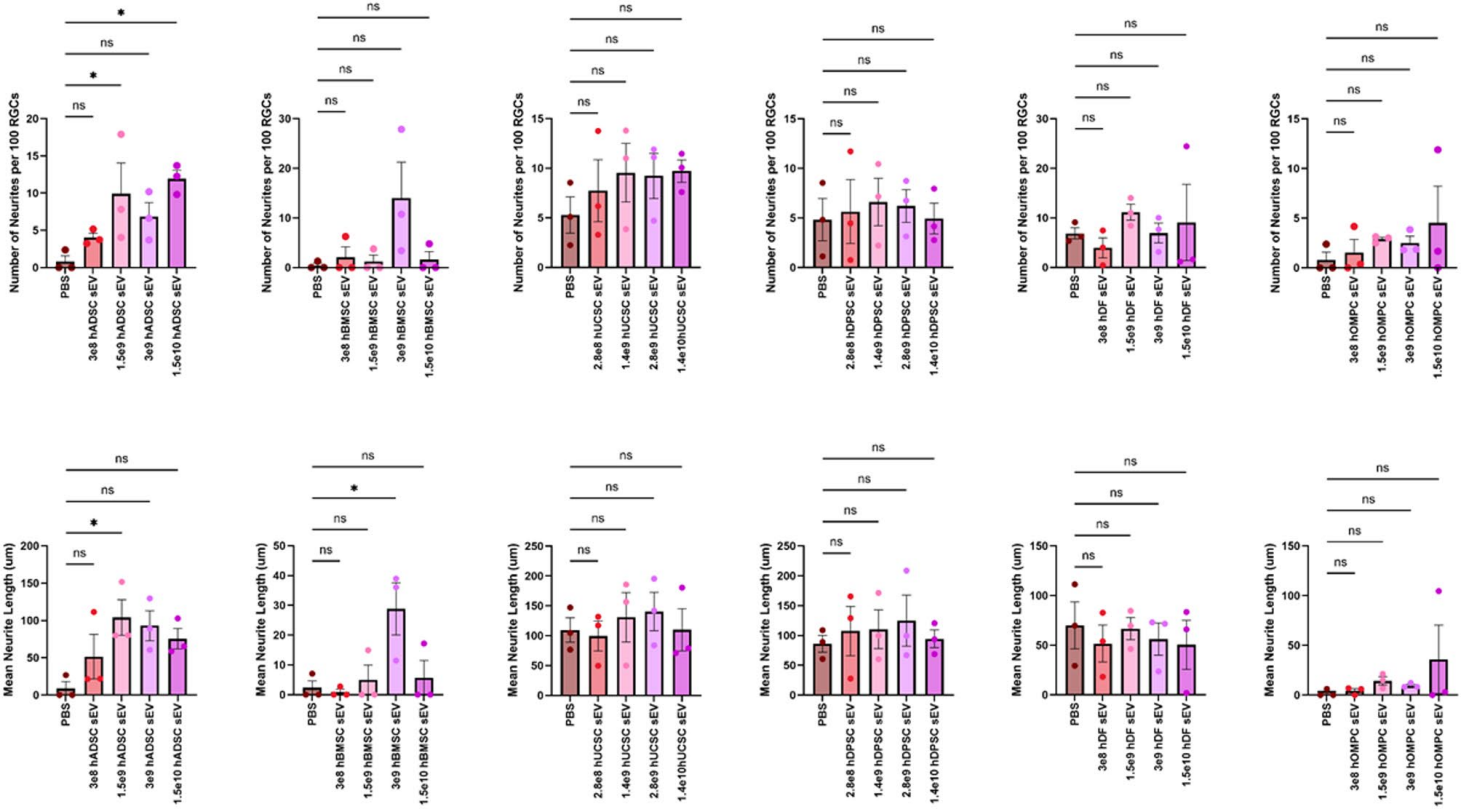
Neuritogenesis in heterogeneous retinal cell cultures treated with small extracellular vesicles (sEVs) from various cell types. Presented here are the number of neurites observed in each well, normalised to 100 cells with RGC-like morphology (top), and the mean length of the 5 longest neurites displayed in µm (bottom). Treatment groups are labelled as the number of sEVs per 350 µl culture medium with error bars showing the standard error of the mean (*n* = 3). Comparisons were carried out using ANOVA. hADSC: human Adipose Stem Cell, hBMSC: Human Bone Marrow Stem Cell, hDPSC: human Dental Pulp Stem Cells, hUCSC: human Umbilical Cord Stem Cell, hDF: human Dermal Fibroblasts, hOMPC: human Oral Mucosal Progenitor Cell, PBS: Phosphate Buffered Saline ** *p* < 0.01; * *p* < 0.05, ns non-significant.

To measure the neuritogenic effect of sEVs, first, the number of neurites observed in each well was counted and normalised to per 100 RGC, then, the five longest neurites were measured and averaged (Fig. 4.). Cell cultures treated with 1.5 × 10^9^ or 1.5 × 10^10^ hADSC sEVs showed a significant increase in neuritogenesis, reaching 9.92 +/- 4.14 (p:0.0405) and 11.93 +/- 1.135 (p: 0.0138) neurites per 100 RGCs respectively, compared to 0.79 +/- 0.79 in PBS treated controls. Although 3 × 10^9^ sEVs were the optimal amount for overall cell or RGC survival, here they did not have a significant influence on neuritogenesis, reaching 6.84 +/- 1.88 (p:0.2059) neurites per 100 RGCs. Similarly, 1.5 × 10^9^ hAMSC sEVs achieved the best results in the analysis of neurite length, reaching 103.9 +/- 23.9 (p: 0.0277) µm on average compared to 93.12 +/- 20.01 μm (p: 0.0508) for 3 × 10^9^ particles, 75.6 +/- 13.7 μm (p: 0.1334) for 1.5 × 10^10^ particles, and 8.94 +/- 8.94 μm for PBS.

Akin to what the data showed for RGC survival rates, hBMSC sEV treatment failed to achieve significant results. However, administering 3 × 10^9^ sEVs noticeably elevated the number of neurites per 100 RGCs to 14.00 +/- 4.94 (p: 0.0633) while the PBS control group had 0.64 neurites per 100 RGCs on average. A great increase in the mean neurite length was also observed for the same treatment group of 3 × 10^9^ hBMSC sEVs, this time reaching the significance level (28.81 +/- 7.49; p: 0.0176) compared to the 2.35 μm average length in the control group.

Although neither of the 4 remaining cell types showed significant influence on neuritogenesis, it is worth noting that hUCSC sEVs increased the number of neurites per 100 RGCs by around 1.8-fold and average length by around 1.2-fold when administered at concentrations of 1.4 × 10^9^ or above. Overall, results suggest the optimal concentration of sEVs to be 1.5 × 10^10^ for increasing the number or 1.5 × 10^9^ for increasing the length of neurites in the case of hADSC, while 3 × 10^9^ vesicles showed the best performance in both metrics for hBMSCs, even though the changed failed to reach significance for the number of neurites observed. sEVs from the other cell types showed very limited positive or negative effect.

## Discussion

In this study, we compared the protective and regenerative potential of the sEVs produced by four types of MSCs, one Fibroblast and one Oral progenitor cell line *in vitro*. Among these, hADSC-sEVs were the most and hOMPC-sEVs the least effective at promoting survival and neuritogenesis in mixed retinal cultures, with hBMSC-sEVs also showing a modest positive effect. Thus, sEVs originating from hADSCs and hBMSCs would be worth further investigating as a potential therapy for glaucomatous nerve damage.

The results shown here are in line with previous studies that showed ADSC-derived EVs to be protective of RGCs and other kinds of neurons. *In vitro*, these sEVs were previously demonstrated to reduce the percentage of apoptotic cells and increase the number of surviving RGCs in retinal cultures subjected to increased hydrostatic pressure^43^. Similarly, positive results have been reported *in vivo* by Ji and colleagues^28^, who showed improvements in RGC survival rates, function, and retinal thickness two weeks after intravitreal injection of sEVs.

BMSCs and their sEVs are the most thoroughly investigated among the cells used in this current study. Given their popularity, their potential has been proven repeatedly demonstrated both *in vitro* and *in vivo.* Indeed, multiple studies have achieved positive results involving human^37,44^ or rodent^12,45,46^ BMSC in various rodent glaucoma models. Among these, the research group of Zheng et al.^43^. showed BMSC-sEVs to protect RGCs from hydrostatic pressure-caused stress *in vitro*, although to a slightly lesser extent than ADSC-sEVs. *In vivo*, intravitreal injections of BMSC-EVs have been shown to protect RGC in various models of glaucoma, including microbead-, laser-, or pressure transducer-induced OHT^14,47^, Optic nerve crush (ONC)^13^, and DBA/2J genetic mice models^48^. In line with previous literature, our observations showed that hBMSC-sEVs could be beneficial *in vitro* if applied at the right concentration.

For hUCSC-sEVs, our results deviate from what has been reported by others. Here, we found no significant benefits on cell survival rates or neurite length, this might be due to the low power and high internal variance of our results or may be explained by UCSC-sEVs acting primarily on supporting cells to encourage RGC survival indirectly. Although not models of Glaucoma, *in vitro* UCSC-sEVs have been shown to protect human retinal vascular endothelial cells (HRECs)^35^ and rat retinal neurons^36^ when cultured in hyperglycemic conditions. In rat ONC models, these vesicles have been reported to be protective of RGCs, without promoting axon regeneration^26^, the opposite of what we observed in vitro. However, yet another study found them to be rather ineffective at preserving RGCs, while also showing their parental cells to be not only protective but also to promote the regeneration of the crushed optic nerve axons^49^.

The EVs of DPSCs, unlike the other MSCs discussed so far, remained relatively unexplored for nervous system or ophthalmological disorders. Their progenitor cells, on the other hand, are well known for their protective and regenerative abilities. *In vitro*, co-culturing human DPSCs with rat retinal isolates was shown to greatly enhance the survival rates of RGCs and to do so in a similar or greater degree than BMSCs or ADSCs, respectively^50^. Our research group also tested these cells *in vivo* in a rat ONC model and found them to preserve retinal thickness, reduce RGC death and promote axon regeneration 21 days after injury^38^. Despite the promising showing of DPSCs in the existing literature, in the current study, their sEVs failed to improve survival rates or axon regeneration.

DF-derived EVs were included due to multiple studies showing them not to be neuroprotective in various glaucoma models, including laser-induced retinal injury^27^, optic nerve crush^13^, and elevated intraocular pressure^14^. However, others found them to promote neurite growth *in vitro*, particularly via activating Wnt10b signalling^51^. In our present study, Fibroblast sEVs performed the worst out of all. Administering these particles appeared to significantly decrease cell survival rates while having a more neutral effect on neurite growth.

Finally, our interest in the EVs of hOMPC arose from the research of Knight and colleagues showing these vesicles to be beneficial for wound healing and to do so more efficiently than hBMSC-sEVs^40^. Although anti-scar-forming properties are less relevant in the context of retinal or neurological diseases, their ability to encourage cell proliferation was considered to be worth exploring. Unfortunately, not only were hOMPC-sEVs observed to be ineffective in promoting neuritogenesis, but they also appeared to be slightly detrimental to cell survival.

Although the results shown here are promising, the conclusions one might draw are limited by the design of our study. First and foremost, RGC cultures are a model of traumatic optic neuropathies, rather than a clinically relevant model of glaucoma or other RGC injuries. The technique involves physically disconnecting the retina from the brain, resulting in the apoptosis of RGCs^52^. With that said, the model was created for testing pharmacological and genetic approaches for promoting axon growth and neuroprotection on mature RGCs, making it perfectly suitable for our purposes. The second major limitation is that RGCs were identified based on morphology, rather than specific protein markers. Although using cell shape and β-III-tubulin distribution is not an uncommon method for identifying RGCs in vitro, β-III-tubulin is also expressed by other neural cells^13,50,53^. Therefore, a more accurate identification could be achieved if Brn3a or RBPMS staining was also included. Finally, the digestion of retinae eliminates some much-needed interactions between RGCs and their neighbouring supporting cells and removes RGCs from the context of an organised tissue. These supporting cells not only play an important role in maintaining neural health but may also take some of the administered sEVs up, providing neuroprotection in an indirect form.

We also did not investigate how certain sEVs achieved their beneficial effects, and why some were better than others. EVs carry a wide variety of cargo molecules and surface proteins, all of which can affect the internal biochemistry of the recipient cell^17,18^. Among these, miRNA garnered special interest, as interfering with their action has been shown to diminish the beneficial effects of EVs^13–16^. Isolating and comparing the miRNA cargo of the EV presented here could lead to a better understanding of their mechanism of action. Furthermore, investigating the other components of MSC secretomes would provide better insight into why certain EVs do not share the protective capabilities of their progenitor cell.

In conclusion, we found sEVs isolated from hADSCs and hBMSCs to be the most effective at promoting survival and neurite growth in rodent RGCs cultures. Moreover, concentrations of 1.5 × 10^9^ – 3 × 10^9^ vesicles per 125.000 cells tended to achieve the best results on both of these metrics. Thus, further investigations may be worth conducting, focusing on either the *in vivo* efficacy of hADSC and hBMSC sEVs, or the mechanism of action responsible for the observed benefits.

## Methods

### Antibodies

**Table:** 

Antibody	Cat. Number	Distributor	Dilution
Anti-β-III-Tubulin	T8578-200UL	Merck	1:400
Alexa Fluor^®^ 488 Goat anti-mouse IgG	A32723	Invitrogen	1:500
Anti-CD81	10630D	Invitrogen	1:500
Anti-CD9	MAB1880	R&D Systems	1:500
Mouse IgG2a kappa Isotype Control	14-4724-85	Invitrogen	1:500
Goat anti-Mouse IgG (H + L), Biotin	31,800	Invitrogen	1:6000

### Mesenchymal stem cell cultures

Human Mesenchymal Stem Cells from Bone Marrow (MSC-031, RoosterBio), Adipose tissue (C46001AD, RoosterBio), Umbilical Cord (V-hUCSC, Newmarket Scientific), or Dental Pulp (PT-5025, Lonza), Human Dermal Fibroblasts, adult (C0135C, Gibco), and human Oral Mucosal Progenitor Cells were seeded into T175 flasks (10127340; Thermo Fisher) at ~ 1.2 × 10^5^ cells/cm2 initial density (~ 2 million cells per flask) and incubated at 37 °C and 5% CO2 in 35 ml culture medium. hADSC, hBMSC, hDF, and hOMPC cells received Dulbecco’s Modified Eagle Medium (DMEM; 61965059; Thermo Fisher) supplemented with 10% EV-depleted fetal bovine serum (FBS; 15434573; Thermo Fisher) and 1% penicillin/streptomycin (P/S). For hUCSCs and hDPSC cells, alpha Minimum Essential Medium (αMEM; 22571038; Thermo Fisher) was provided similarly supplemented with 10% FBS and 1% P/S, with hUCSC cells also receiving Basic fibroblast growth factor (bFGF, 78003.1; STEMCELL Technologies) as per the supplier’s instructions. The medium was replaced every 2–3 days, and cells were passaged when over 80% confluent using 0.25% trypsin/ EDTA (11590626; Thermo Fisher). Except for the hOMPC cell line, cells were used at passages 2–5 post-acquisition.

### Extracellular vesicle isolation and storage

sEVs were isolated using differential ultracentrifugation. Briefly: Culture medium was sequentially centrifuged at 300xg for 10 min, 2,000xg for 10 min and 10,000xg for 30 min, before being forced through a 0.22 μm pore size Millex^®^-OR Filter Unit (SLGP033RS; Sigma-Aldrich). The supernatant was collected, and pellets were discarded in each step. Filtered supernatant was spun at 100,000xg for 70 min. Pellets were collected, pooled, and washed in large amounts of sterile PBS before being spun down a second time at 100,000xg for 70 min. A constant temperature of 4 °C was maintained for each spin. Pellets were collected in 1–2 ml sterile PBS and condensed down to less than 100 µl using Amicon™ Ultra-4 Centrifugal Filter Units (UFC8100; Millipore) equilibrated with Milli-Q water prior to use as advised by the manufacturer. Isolates were stored at -80 °C.

### Extracellular vesicle quantification and quality control

For quantification, a ZetaView^®^ Nanoparticle Tracking Analyser (Particle Metrix GmbH, Ammersee, Germany) was used. Particle size and concentration were measured at 11 positions over 1 cycle (Laser Wavelength: 488 nm; Filter Wavelength: Scatter; Sensitivity: 80.0; Shutter: 100). Time-resolved fluorescence TRF was used for further confirmation, targeting the known exosomal markers CD81 and CD9, as well as mouse IgG for a negative control. Protein concentration of sEVs samples was measured using a Micro BCA™ Protein Assay Kit (23235; Thermo Scientific™) according to the manufacturer’s instructions. Equal amounts of protein (1 µg/well) were transferred to a high-binding, flat-bottom 96-well plate (655097; Greiner), topped up to 300 µl total volume with PBS, sealed, and incubated overnight at 4 °C. The following day, the samples were blocked in 10% milk diluted in PBS + 0.1% Tween-20 (P1379; Sigma) for 2 h, and incubated with 1 µg/well primary, and 0.2 µg/ml biotin-conjugated secondary antibodies for 1 h in 100 µl buffer (Reagent Dilutent Concentrate 2 diluted 1:5 in distilled water, DY995; R&D Systems). This was followed by a further 45-minute incubation with Europium-labelled Streptavidin (1244-36; DELFIA) diluted to 0.1 µg/ml in RED Buffer (42 − 02; Uniogen Oy) and another 10 min in a Fluorescence enhancer solution (1244 − 105; DELFIA). Samples were washed 3 times between each step and 6 times before the fluorescent enhancer was added (Wash Buffer: Reagent Dilutent Concentrate 2 diluted 1:100 in distilled water, DY995; R&D Systems). All incubation steps were carried out at room temperature with shaking while the plates were sealed. Fluorescence was measured using a microplate reader (FLUOstar Omega; BMG Labtech).

### Animals

Animals were kept and euthanised according to local guidelines. Ethical approval was obtained from the Cardiff University’s Animal Welfare Ethical Review Body (AWERB). All protocols and methods were conducted in accordance with local guidelines and pre-approved standard operating procedures. Adult, female, Sprague Dawley Rats weighing 100–150 g were supplied by Charles River Laboratories. Constant surveillance was provided by trained staff under conditions of 21 °C, 55% humidity and 12-12-hour light and dark cycle, with food/water given *ad libitum*. Euthanasia was carried out via rising concentrations of CO2, without prior anaesthesia. 100% CO2 gas was delivered at a rate of 10 L/min for 4 min and 14 L/min for a further 4 min. Death was then confirmed via cervical dislocation and harvested tissues were immediately processed.

### Retinal ganglion cell cultures

Retinal cells were isolated using a Papain Dissociation kit (PDS/LK003150; Worthington Biochemical). Briefly: retinae were dissected, minced and incubated in a solution of Papain and DNase diluted in EBSS at 37 °C for 90 min with constant motion. Digested tissue was then centrifuged for 6 min at 300xg, the supernatant was discarded, and the pellet was resuspended in a mixture of 1.35 ml EBSS, 150 µl Albumin Ovomucoid inhibitor, and 75 µl DNase. This suspension was carefully transferred onto 2 ml Albumin Ovomucoid inhibitor, creating a density gradient, and centrifuged at 70xg for 6 min. The supernatant was discarded and pelleted cells were resuspended in 1 ml supplemented Neurobasal Plus medium (Neurobasal-A [A3582901; Thermo Fisher] 2% B27 supplement [17504044; Gibco], 0.5 mM L-glutamine [25030081; Thermo Fisher] and 50 mg/ml of gentamycin [15710064; Thermo Fisher]).

125,000 retinal cells were seeded in 300 µl Supplemented Neurobasal Plus medium in each of eight wells of Millicell^®^ EZ Slides (PEZGS0816; Millipore) at ~ 1.8 × 10^5^ cells/cm2 pre-coated with 100 mg/ml poly-D-lysine (A3890401; Thermo Fisher) for 60 min and 20 mg/ml laminin (10267092; Fisher Scientific Ltd) for 30 min. Cultures were immediately treated with sEVs resuspended in 50 µl PBS or PBS alone (Final volume: 350 µl per well) and incubated for 3 days under conditions of 37 °C and 5% CO_2_.

### Immunocytochemistry

Retinal cell cultures were fixed in 4% Polyformaldehyde (PFA) for 10 min and blocked in 3% Bovine Serum Albumin and 0.1% Triton-X 100 (T9284-500ML; Sigma-Aldrich) solution for 20 min. Fixed cultures were then incubated for with anti-β-III-Tubulin primary and Alexa Fluor^®^ 488 Goat anti-mouse fluorescent secondary antibodies for 1 h at room temperature in 3% Bovine Serum Albumin, 0.05% Tween 20 (P1379-500 ml; Sigma) solution.

For mounting, VECTASHIELD^®^ Antifade Mounting Medium containing DAPI (H-1200-10; 2B Scientific) was used. Samples were washed in PBS for 10 min, 3 times between each step. Mounted slides were left to dry overnight, and stored at 4 °C shielded from light.

### Fluorescent microscopy

Samples were visualised using a Leica fluorescent microscope system (Leica DM6000 body, DFC350 FX monochrome Digital Camera, EL6000 External light source) at 600x magnification with excitation and emission wavelengths of 358/461 nm and 493/519 nm for DAPI and AlexaFluor488, respectively.

DAPI-stained cells, RGCs, and neurites were counted manually. Here, RGCs were defined as elliptical cells where β-III-tubulin forms a halo around the nucleus, with a pole of stronger clustering also observable.

### Data analysis

Images were collected of 10–15 neurites per well (where possible) and their length was determined using the Fiji Neuroanatomy Plugin as described by Durmaz and colleagues^54^.

Data was analysed and visualised using GraphPad Prism 10 Software. Treatment groups were compared to PBS control using one-way ANOVA with Dunnett test. (* *p* > 0.05; ** *p* < 0.01; *** *p* < 0.005).

Post-hoc Power calculation was carried out using R Studio’s pwr package (**pwr.anova.test(k = 5**,** f = Effect_size**,** sig.level = 0.05**, *n*** = 3**). Effect size (Cohen’s f) was determined using a free online calculator (Effect Size Calculators; Partial eta-squared (Fixed effects); https://effect-size-calculator.herokuapp.com/#partial-eta-squared-fixed-effects*).*

## Data Availability

All data created and examined throughout this investigation are contained within this article. Upon request, the corresponding author will provide any datasets used or analyzed during the current work.
